# Atherogenic Index of Plasma (AIP) a Tool to Assess Changes in Cardiovascular Disease Risk Post Laparoscopic Sleeve Gastrectomy

**DOI:** 10.1155/2020/2091341

**Published:** 2020-08-01

**Authors:** Eman Al Shawaf, Ebaa Al-Ozairi, Fahad Al-Asfar, Anwar Mohammad, Shaima Al-Beloushi, Sriraman Devarajan, Fahd Al-Mulla, Jehad Abubaker, Hossein Arefanian

**Affiliations:** ^1^Department of Biochemistry and Molecular Biology, Dasman Diabetes Institute, Kuwait; ^2^Medical Division, Clinical Research Unit, Dasman Diabetes institute, Kuwait; ^3^Departement of Surgery, Faculty of Medicine, Kuwait University, Kuwait; ^4^Department of Immunology and Microbiology, Dasman Diabetes Institute, Kuwait; ^5^National Dasman Diabetes Biobank, Dasman Diabetes Institute, Kuwait; ^6^Research Division, Dasman Diabetes Institute, Kuwait

## Abstract

Predictive indices like the atherogenic index of plasma (AIP) have been developed to estimate the risk of cardiovascular disease (CVD). Metabolic surgery is the most effective treatment for a rapid improvement of morbid obesity and its comorbidities such as type 2 diabetes (T2D) and CVD. A decreased reoccurrence of CVD after metabolic surgery has been reported by several studies. However, studies utilizing predictive indices for CVD risk in CVD-free morbid-obese patients who undertook laparoscopic sleeve gastrectomy (LSG) are lacking. Here, we use AIP as a tool to evaluate the improvement in CVD risk post-LSG in morbid-obese people who had no history of CVD. *Method.* We compared baseline, 6- and 12-month post-LSG score of AIP, vascular age, circulating biochemical markers related to CVD in two groups of BMI and age-matched morbid-obese participants with and without T2D. *Results.* At baseline, people with T2D had significantly higher AIP both, with morbid obesity (0.23 ± 0.06, *p* < 0.001) and normal weight (0.022 ± 0.05, *p* < 0.001) compared to their BMI-matched without T2D group. People with morbid obesity had low AIP (−0.083 ± 0.06). Vascular age was significantly higher in people with morbid obesity and T2D (65.8 ± 3.7year, *p* < 0.0001) compared to morbid obesity (37.9 ± 2.6 year). After one year, AIP was significantly reduced compared to baseline score in people with morbid obesity with/without T2D, respectively (−0.135 ± 0.07, *p* = 0.003; and −0.36 ± 0.04, *p* = 0.0002). *Conclusion*. Our data illuminates AIP as a reliable predictive index for CVD risk in morbid-obese people who had no history of CVD. Moreover, AIP accurately distinguishes between morbid obesity with T2D and morbid obesity and showed a rapid and significant reduction in CVD risk after LSG in people who had no history of CVD. This is a ClinicalTrials.gov registered trial (Reference NCT03038373).

## 1. Introduction

Obesity is a global health burden that is associated with a number of abnormalities and comorbidities, most notably insulin resistance, dyslipidemia, hypertension [1], a higher risk of developing type 2 diabetes (T2D), and cardiovascular disease (CVD) [2]. The past years involved a rise in metabolic surgeries that emerged as a rapid and effective treatment option for people with severe obesity. These surgeries improved weight and resolved obesity-related comorbidities through correcting various mechanisms/factors such as oxidative stress and inflammation [3–5].

Elevated levels of a proinflammatory molecule, such as c-reactive protein (CRP), contribute to a rise in oxidative stress and consequently lipid peroxidation. Oxidative stress is one of the factors contributing to vascular dysfunction, which is implicated in the pathogenesis of atherosclerosis, T2D, and hypercholesterolemia [6, 7]. Lipid peroxidation is a potential key event in pathological processes affecting the integrity of the vascular wall [8, 9]. In addition to this, dyslipidemia is another risk factor for premature atherosclerosis and an increased CVD risk. The significance of dyslipidemia is based on the triad of an increase in low-density lipoprotein (LDL) and triglycerides (TG) particles and a decrease in high-density lipoprotein (HDL) levels [10]. To assess patients' risk of developing CVD, various novel biomarkers have been identified [11]. The use of biomarkers in clinical investigation has a prognostic value, as it helps in an early identification of CVD at-risk patients [11–13]. Conventional risk factors for CVD helped in developing various risk prediction models, which had and continues to hold great importance. However, there is a considerable percentage of patients who do not show the traditional risk factors [11]. Additionally, quantitative measurement of LDL subpopulations was introduced as an effective way for CVD risk stratification, but the current analytical methodologies lack standardization and clear clinical relevance [14]. For which identifying novel biomarkers and developing new indices that are accurate, reliable and easy to quantify is required.

The atherogenic index of plasma (AIP) is a calculated index that strongly reflects the future risk of atherosclerosis and CVD and predicts atherogenicity [15–17]. AIP which is the logarithm of plasma TG to HDL ratio has shown a direct and independent association with arterial stiffness [17]. Considering the factors used to calculate AIP, this index reflects the opposing influence of TG and HDL on inflammation, oxidative stress, extracellular matrix formation, and the phenotypic changes affecting vascular smooth muscle [18, 19]. In this study, our aim was to assess the use of AIP in predicting CVD risk in morbid-obese people who had no history of CVD before and after laparoscopic sleeve gastrectomy (LSG). Furthermore, we used AIP to identify the time required for LSG to induce a significant change in the patients' CVD risk.

## 2. Materials and Methods

### 2.1. Study Population

The study included two main groups of people, individuals with morbid obesity who underwent LSG as a surgical intervention, and people with normal weight as control. The group with morbid obesity included people with T2D (*n* = 17, mean age 39 ± 2.5 years), and people without T2D (*n* = 23, mean age 32 ± 2.6 years). As described previously [20], from participants who underwent LSG, only 11 with T2D and 17 without T2D were followed up for one year after surgery (i.e., after 6 months and 1 year). The control groups included normal-weight participants that were not exposed to the surgical intervention. This included normal-weight people with T2D (*n* = 19) and normal-weight people without T2D (*n* = 15) who had no previous or current medical conditions. Exclusion criteria in this study were having a previous metabolic surgery, type 1 diabetes mellitus, T2D>5-years, insulin replacement therapy, CVD history, malignancy, and pregnancy. Power analysis for the study was calculated using G-Power software, version 3.1.9.6 [21, 22], to calculate the effective sample size required for our cross-sectional study. The total sample size requirement was calculated with a statistical power (1-*β*) of 0.09, *α* of 0.05, and an effective size of 0.80, whereby the minimum size was 34 participants in each group to test the hypothesis at *p* < 0.05. Recruitment process congregated 40 participants with morbid obesity and 34 participants with normal weight. Additionally, participants were classified depending on the presence/absence of T2D into 36 participants with T2D and 38 participants without T2D. This study was approved by the Scientific Advisory Board and Ethical Review Committee at Dasman Diabetes Institute (DDI). Both verbal and written consents were obtained from all participants. This is a ClinicalTrials.gov registered trial (Reference NCT03038373).

### 2.2. Anthropometric and Biochemical Measurements

As described [20], body measurements including weight, height, hip, and waist circumference were taken by a trained nurse. Blood pressure (BP) values are the average of three consecutive measurements. Body composition analysis was performed using the IOI 353 body composition Analyzer (Jawon Medical, Korea). The diagnostic criteria were based on ADA classification [23], according to which participants were classified as people with diabetes when having fasting plasma glucose ≥7.0 mmol/L, 2-hr postprandial plasma glucose ≥11.1 mmol/L, A1c ≥6.5%, or having a random blood glucose level ≥11.1 mmol/L in the presence of signs and symptoms of diabetes. Circulating biochemical parameters were quantified in peripheral venous blood samples collected from participants following an overnight fast, a minimum of 10 hours. Presurgery, baseline blood samples were collected from all participants. Follow-up visits involved a similar procedure of taking body composition and collecting fasting blood samples. Fasting insulin levels were determined using the Access Ultrasensitive Insulin Assay (Beckman Coulter, Brea, CA); c-peptide was determined by the DiaSorin LIAISON® analyzer. Fasting plasma glucose (FPG), serum total cholesterol (TC), LDL, HDL, TG, albumin, alanine aminotransferase (ALT), aspartate aminotransferase (AST), and high-sensitivity c-reactive protein (hsCRP) were measured by the Siemens Dimension RXL Max Integrated Chemistry analyzer (Diamond Diagnostics, Holliston, MA). A1c was measured using Variant™ (Bio-Rad, Hercules, CA). All clinical tests were performed by the clinical laboratory of Dasman Diabetes Institute as routine clinical diagnostic tests. In our research lab, plasma was collected from blood samples, aliquoted, and stored at -80°C for further analysis. Levels of oxidized low-density lipoprotein (oxLDL) were quantified through a commercial human ELISA kit (SEA527Hu; Wuhan USCN, China) following manufacturer's protocol with an intra-assay CV <10% and an interassay CV <12% (provided by the manufacturer).

### 2.3. Calculations

Body mass index, BMI (kg/m^2^), was calculated as the body weight (in kilograms)/height (in square meters). Hip and waist circumference values were used to calculate the waist-hip ratio. AIP was calculated as the base 10 logarithm of the ratio of (TG/HDL) [15]. An AIP value <0.11 is associated with a low risk of CVD; AIP value from 0.11 to 0.24 indicates an intermediate CVD risk, while AIP ≥0.24 suggests an increased CVD risk [15, 16]. Framingham CVD risk equation was used to calculate an estimate of participants' vascular age [24]. In this mathematical model, various parameters are considered including age, sex, diabetes, systolic blood pressure, smoking, TC, and HDL levels.

### 2.4. Statistical Analysis

Statistical analysis was performed as previously described [20]. Briefly, normal distribution was tested using the Shapiro-Wilk normality test. Differences between the study groups at baseline were determined by a two-tailed unpaired *t*-test for normally distributed parameters. Data not following normal distribution was analyzed with nonparametric tests, Mann–Whitney *U* and Wilcoxon test, as appropriate to evaluate the difference between study groups at baseline. Analysis of variance (ANOVA) was used to compare pre- and postsurgery data and Bonferroni test for multiple comparisons to determine statistical significance. Data are presented as mean ± SEM, or median with interquartile range, with a *p* value ˂0.05 indicating significance. Statistical analysis was performed using GraphPad Prism 6 software (GraphPad software, Inc., CA, USA) and SPSS version 25 (SPSS Inc., Chicago, IL, USA).

## 3. Results

Table 1 summarizes the anthropometric characteristics and metabolic variables of the study population at baseline. Levels of hs-CRP were elevated in people with morbid obesity and T2D (1.3 ± 0.1 mM, *n* = 17) and without T2D (1.1 ± 0.1 mM, *n* = 23), reflecting a state of inflammation that suggests a possible moderate CVD risk in participants with morbid obesity, both with and without T2D. At baseline, lipid profile of people with morbid obesity and T2D was characterized by increased levels of TG (median: 1.54, 1.07-2.02, *n* = 17), and very-low-density lipoprotein (VLDL; 0.7 ± 0.08 mM, *n* = 17) and reduced HDL (0.9 ± 0.04 mM, *n* = 17) that was significantly different from people with morbid obesity (Table 1).

### 3.1. Diabetes Is a Critical Factor in Determining AIP and the Vascular Age of Patients

**Figure 1:**
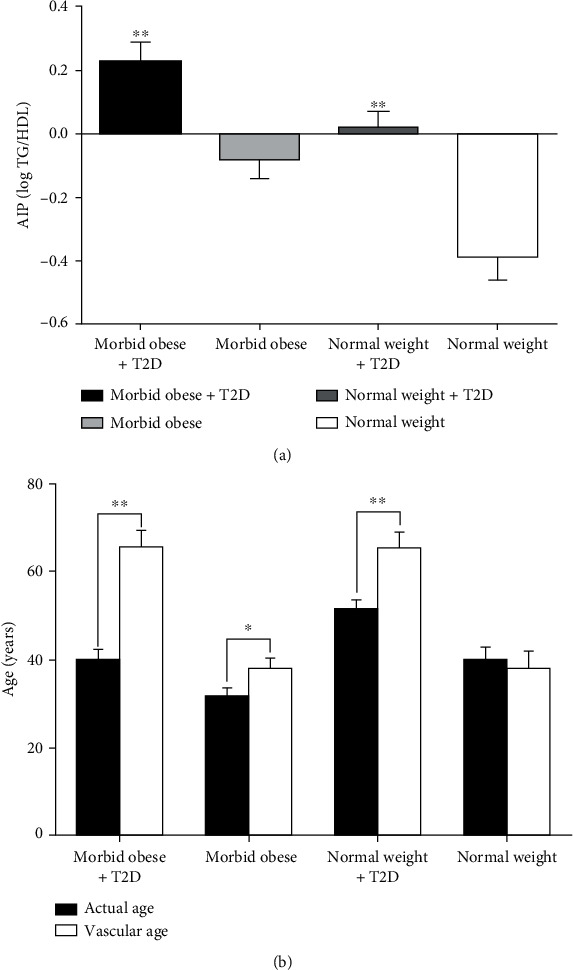
Baseline data calculation of (a) Atherogenic index of plasma (AIP) score in all study groups. Participants with morbid obesity and T2D (*n* = 17) show a significantly higher AIP (0.23 ± 0.06, *p* < 0.0001), compared to morbid obesity alone (*n* = 23, −0.083 ± 0.06). In participants with normal-weight and T2D (*n* = 19), the calculated AIP (0.022 ± 0.05) reflecting a lower CVD risk was compared to participants with morbid obesity and T2D. Black, light gray, dark gray, and white bars represent calculated baseline AIP of morbid obese + T2D, morbid obese, normal weight + T2D, and normal weight groups, respectively. (b) The estimated vascular age (Framingham score) has a significant rise in participants with T2D from both weight groups morbid obese (65.8 ± 3.7 year), and normal weight (65.6 ± 3.6 year, *p* < 0.0001) compared to their actual age. Participants with morbid obesity showed a significant difference between the vascular age (37.9 ± 2.6 year, *p* < 0.001) and chronological age. There was no significant difference between the vascular age and the actual age of people with normal weight. Black and white bars represent actual age and estimated vascular age, respectively, in each group. ^∗^*p* < 0.001, and ^∗∗^*p* < 0.0001.

At baseline, the AIP showed that (1) people with T2D had elevated levels of AIP (i.e., both morbid obese and normal weight) compared to participants without T2D; (2) AIP was significantly higher in people with morbid obesity and T2D (0.23 ± 0.06 log TG/HDL, *n* = 17, *p* < 0.001) compared to BMI-matched people (−0.083 ± 0.06 log TG/HDL, *n* = 23), and normal-weight participants with T2D (0.02 ± 0.05 log TG/HDL, *n* = 19, *p* < 0.001) compared to control (−0.39 ± 0.07 log TG/HDL, *n* = 15); (3) normal-weight participants with T2D had lower AIP score (0.022 ± 0.05 log TG/HDL, *n* = 19), indicating a lower CVD risk compared to participants with morbid obesity and T2D. This suggests T2D as an essential factor for higher AIP score; however, T2D combined with obesity would exacerbate the condition leading to a significant increase in AIP score and consequently elevated CVD risk (Figure 1(a)). Quantification of plasma oxLDL showed that people with morbid obesity and T2D had the highest levels of oxLDL (1.7 ± 0.17 mg/mL, *n* = 17, *p* < 0.01) compared to other groups (Table 1).

There was a significant increase in the estimated vascular age in comparison to the actual age at baseline (Table 1). The comparison between the actual age and the calculated vascular age showed participants with morbid obesity and T2D to have the highest mean difference 26 ± 2.5 years (95% CI: 20.6–31.28, *p* < 0.001). People with normal weight and T2D showed the second-highest difference between vascular age and their actual age, with a mean difference 14 ± 3, (95% CI: 8.27–21.06, *p* < 0.01). Finally, people with morbid obesity had the lowest mean difference 6 ± 1.5 years (95% CI: 3.11–9.75, *p* < 0.001). There was no significant difference between the actual age and the vascular age in people with normal weight (Figure 1(b)).

### 3.2. LSG Reduces the Risk of CVD and Improves Vascular Age of Patients

Post-LSG participants displayed a rapid improvement in various clinical parameters. LSG ameliorated FPG, A1C, c-peptide, insulin resistance (iHOMA-IR), and *β*-cell activity (iHOMA2% *β*), indicating T2D remission (previously reported [20]). There was a gradual decline in weight loss post-LSG that was reflected by BMI, excess weight loss (%EWL), and body fat (%BF) in people with morbid obesity, with and without T2D, which was significant after one year of LSG [20]. Lipid metabolism improved significantly post-LSG, and VLDL levels dropped significantly after 6 months (0.48 ± 0.07 mM, *n* = 10, *p* < 0.05) and 1 year (0.39 ± 0.06 mM, *n* = 10, *p* < 0.01) of surgery in people with morbid obesity and T2D. There was no significant difference between levels of oxLDL at baseline and 1-year post-LSG, in people with morbid obesity (i.e., with and without T2D) (Table 2).

**Figure 2:**
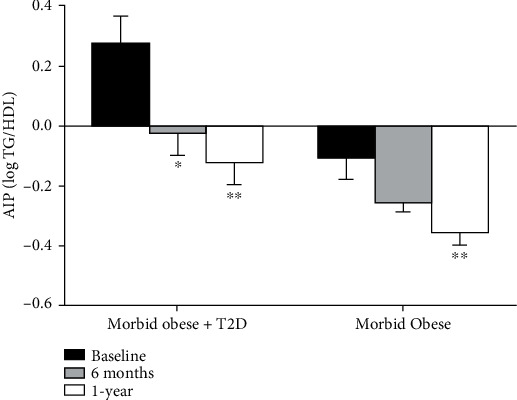
Improvement in AIP score post-LSG. There is a significant drop in AIP score in participants with morbid obesity and T2D, *n* = 11 after 6 months (−0.03 ± 0.07, *p* < 0.01) and after 1 year of LSG (−0.13 ± 0.07, *p* < 0.01) compared to their baseline levels. In people with morbid-obesity (*n* = 17), AIP decreased after 6 months of LSG (−0.26 ± 0.03), and the change was significant only after 1 year of LSG (−0.36 ± 0.04, *p* < 0.001) compared to the baseline levels. Black, gray, and white bars represent calculated AIP at baseline, 6-month, and 1-year post-LSG, respectively. ^∗^*p* < 0.01, and ^∗∗^*p* < 0.001.

In general, there was a gradual decline in AIP score post-LSG (Figure 2). People with morbid obesity and T2D showed a significant reduction in AIP score after 6 months of LSG (−0.03 ± 0.07 log TG/HDL, *n* = 11, *p* < 0.05), and AIP was further reduced after 1 year (−0.13 ± 0.09 log TG/HDL, *n* = 11, *p* < 0.01). AIP in people with morbid obesity significantly changed after 1 year of surgery (−0.36 ± 0.04 log TG/HDL, *n* = 17, *p* < 0.01). Furthermore, we report a prominent decline in the calculated vascular age in both study groups after 1 year of LSG. The difference between the actual age and the calculated vascular age decreased by 23% in people with morbid obesity and T2D, and it dropped by 6.5% in people with obesity. Consequently, there was no significant statistical difference between the actual age and the calculated vascular age of participants 1 year after LSG (Table 2).

## 4. Discussion

Obesity on its own or when combined with T2D introduces a range of physiological and biochemical abnormalities that predispose to the development of CVD. Many studies have reported the relationship between obesity, inflammation, and CVD and its consequences on vascular structure and function, which leads to the development of atherosclerosis [25]. As such, an early detection of atherosclerosis at the subclinical period is important. In clinical settings, plasma lipoprotein profile is used as an indicator of cardiovascular risk; however, recent studies have reported the atherogenic index of plasma as a better marker of atherosclerosis and arterial stiffness [17, 26].

In the present study, we used AIP as a tool to evaluate the vascular health of participants concomitantly with the conventional biomarkers. The measurements were taken at baseline and subsequently after LSG, to ascertain the improvement in the vascular health of individuals with obesity and T2D. From our results, T2D was associated with elevated AIP, and AIP score exacerbated when T2D was combined with morbid obesity (Figure 1(a)). The AIP results corroborated with the vascular age data, whereby the greatest increase occurred in individuals with T2D and morbid obesity combined. Additionally, independent of obesity, T2D was associated with an increase in the vascular age (Figure 1(b)). Therefore, our data supports the use of AIP as a method to reflect vascular health and potential CVD risk.

Our baseline data showed that people with morbid obesity (i.e., with and without T2D) have a significant increase in inflammation compared to people with normal weight (Table 1). On the other hand, people with T2D showed an abnormal lipid profile, and this is independent of BMI. In our study, people with T2D, both with morbid obesity and normal weight, had a significant increase in TG and VLDL levels and a significant reduction in HDL levels compared to those without T2D (Table 1). In people with T2D, hypertriglyceridemia occurred secondary to having high levels of VLDL, small dense LDL (sdLDL), and low HDL levels [27, 28], which are known to hold atherogenic traits [29]. Henceforth, TG levels would serve as a predictive marker for atherosclerosis and potential CVD. Moreover, sdLDL particles were reported with a significant role in the development of arteriosclerosis and CVD [28]. Therefore, the importance of AIP as an index to detect premature atherosclerosis [30] came from its correlation with sdLDL particles [31], and its inverse correlation with LDL particle size [32].

Considering the importance of oxLDL molecules in systemic inflammation and oxidative stress [33], we quantified levels of circulating oxLDL. In our study, oxLDL was elevated in people with morbid obesity and T2D, in comparison to all other groups (Table 1). Elevated levels of circulating oxLDL have been associated with obesity, hyperglycemia, and hypertriglyceridemia [34]. Nonetheless, changes in oxLDL levels after LSG were not significant in comparison to baseline levels (Table 2). The absence of a significant change in LDL oxidation in our results might be due to other factors related to the study population. The study was limited to people who had been diagnosed with T2D <5 years and had no history of CVD. This might indicate that specific pathological conditions are required to exacerbate the presence of oxLDL at which it would serve as a marker of inflammation and atherosclerosis, which were not attained in our study population. On the other hand, the association between oxLDL and metabolic syndrome is established, where weight loss was found to reduce oxLDL [35, 36]. Thus, our oxLDL data must be interpreted with caution.

Baseline AIP was elevated in people with T2D compared to those without diabetes. Additionally, people with both obesity and T2D had the highest AIP score suggesting an intermediate risk of CVD, compared to people with a normal weight and T2D. In the absence of T2D, people with obesity and normal weight had low AIP values indicating a negligible adverse effect on vascular health (Figure 1(a)). This was emphasized in a recent report where having a combination of low HDL levels and high TG accompanied by reduced insulin sensitivity defined atherogenic dyslipidemia [37]. Additionally, we used Framingham's CVD Risk Predictive formula as a method to estimate the vascular age of participants [38, 39]. Our data showed a significant rise in the calculated vascular age of people with T2D and morbid obesity (Figure 1(b)), which accentuated the deleterious effects of T2D and dyslipidemia on vascular health. Independent of T2D, there was a significant difference between the actual age and the calculated vascular age of participants with morbid obesity; however, the difference was less prominent compared to the combined effect of T2D and obesity.

AIP is an independent risk factor of coronary artery disease, which has been associated with the lipoprotein particle size and was found to have an inverse correlation with LDL particle size [17]. AIP is reported to have a predictive potential for hypertension, diabetes, and vascular events [16, 31] as it reflects the health of the arteries through a number of processes involved in arterial stiffness such as inflammation, oxidative stress, extracellular matrix formation, and vascular smooth muscle phenotype change. Dobiasova et al. reported a significant correlation between AIP and the number of total large and medium-sized VLDL particles [16]. Additionally, AIP was significantly associated with the atherogenic apoB and the atheroprotective ApoAI molecules [16]. Considering the importance and efficacy of AIP in predicting and evaluating CVD risk [15–17, 31], we utilized it to evaluate the effect of LSG on patients' vascular health. Our data showed a significant improvement in AIP score post-LSG, which indicated an improvement in the vascular health of people with morbid obesity and T2D. The improved vascular health came secondary to an improved lipid metabolism, insulin sensitivity, and T2D remission after LSG. Therefore, an easily calculated index as AIP would be a valuable tool to monitor the efficacy of metabolic surgery on vascular health. It is noteworthy to highlight that our study participants were diagnosed with T2D for a relatively short time span (i.e., ≤5 years). This was a critical factor to limit and avoid the occurrence of nonreversible T2D complications, and consequently allow T2D remission.

Our study displayed several limitations, including the small sample size combined with the difference in men/women ratio, the lack of a detailed analysis of LDL population, and a relatively reduced follow-up rate in some post-LSG visits due to unexpected circumstances leading to participants unavailability. This is a major limitation for which our data should be interpreted with caution. A similar study on a larger scale that would consider including other parameters as a direct measurement of arterial stiffness (i.e., pulse wave velocity) would be expected to give a better understanding of the importance of AIP as a simple tool to evaluate the rapid improvement in vascular health after metabolic surgery procedures.

## 5. Conclusion

Our AIP data showed T2D as an important factor with a potential effect on vascular health. AIP score was worsened with the combined effect of T2D and morbid obesity. AIP as a reflection of vascular health, accurately made the distinction between the conditions, T2D, morbid obesity or T2D, and morbid obesity combined. Additionally, AIP showed the rapid improvement post-LSG indicating a quick reversion and restoration of cardiovascular health. Thus, AIP would be an alternative to complex and expensive methods that require measuring LDL subpopulations (e.g., using filtration chromatography) and determination of sdLDL concentration to evaluate biomarkers atherogenic potential in patients.

## Figures and Tables

**Table 1:** Clinical characteristics of study participants subjects at baseline.

Variable	Morbid obese		*p* value	Normal weight		*p* value
	With T2D *N* = 17	Without T2D *N* = 23		With T2D *N* = 19	Without T2D *N* = 15	
Age (year)	39.9 ± 2.4	31.5 ± 2.2		51.6 ± 2.1	39.9 ± 2.9	0.008^∗^
Weight (kg)	121.0 ± 5.2	120.7 ± 4.8		67.4 ± 2.2	66.0 ± 3.0	
Height (cm)	166 ± 1.6	164.7 ± 2.2		166.2 ± 2.4	164.8 ± 2.2	
BMI (kg/m^2^)	43.8 ± 1.5	44.6 ± 1.6		23.8 ± 0.2	23.5 ± 0.3	
Body fat (%)	42.8 ± 1.1	44.4 ± 1.0		28.6 ± 1.2	29.0 ± 1.0	
Abdominal circumference (cm)	128.6 ± 2.9	125.0 ± 2.9		96.0 ± 1.7	89.3 ± 2.0	
Waist/hip ratio	0.94 ± 0.02	0.87 ± 0.02		0.95 ± 0.01	0.8 ± 0.02	0.01^∗^
SBP (mmHg)	127.9 ± 3.2	125.4 ± 2.6		125.7 ± 2.8	116.3 ± 4.6	
DBP (mmHg)	81.2 ± 2.2	76.5 ± 1.9		77.6 ± 2.4	71.3 ± 2.8	
Vascular age (year)	65.0 ± 3.0	37.0 ± 2.0	0.0001^∗∗^	65.0 ± 3.0	38.0 ± 4.0	0.0001^∗∗^
TC (mM)	4.8 ± 0.3	4.9 ± 0.1		4.4 ± 0.2	4.8 ± 0.2	
TG (mM)	1.54 (1.07-2.02)	0.92 (0.71-1.65)	0.01^∗^	1.29 (0.91-1.73)	0.66(0.45-0.82)	0.001^∗∗^
LDL (mM)	3.1 ± 0.2	3.1 ± 0.1		2.6 ± 0.2	2.9 ± 0.2	
HDL (mM)	0.9 ± 0.05	1.2 ± 0.1	0.003^∗∗^	1.2 ± 0.05	1.6 ± 0.1	0.0008^∗∗^
VLDL (mM)	0.7 ± 0.09	0.4 ± 0.05	0.025^∗^	0.5 ± 0.045	0.3 ± 0.04	0.056
oxLDL (mg/mL)	1.7 ± 0.17	0.9 ± 0.09	0.007^∗∗^	1.16 ± 0.23	1.18 ± 0.23	
AIP (log TG/HDL)	0.23 ± 0.06	−0.08 ± 0.06	0.0001^∗∗^	0.02 ± 0.05	−0.39 ± 0.07	0.0006^∗∗^
hsCRP (mg/dl)	1.4 ± 0.2	1.1 ± 0.1		0.27 ± 0.07	0.3 ± 0.07	
Insulin (pg/mL)	2092 ± 202	1210 ± 126	0.0004^∗∗^	650 ± 112	350 ± 63	0.03^∗^
c-peptide (pg/mL)	2407 ± 207	1824 ± 135	0.01^∗^	1064 ± 118	875 ± 75	
FPG (mM)	10.7 ± 0.6	5.4 ± 0.08	0.0001^∗∗^	10.0 ± 0.5	5.5 ± 0.1	0.0001^∗∗^
A1c (%)	8.6 ± 0.3	5.8 ± 0.1	0.0001^∗∗^	8.5 ± 0.4	5.5 ± 0.1	0.0001^∗∗^
ALT (IU/L)	51 (39.5-67)	43 (35-56)		42 (38-54)	34 (32-39)	0.005^∗∗^
AST (IU/L)	25 (19.5-36)	20 (16-30)		18 (16-22)	19 (16.75-20.5)	
Sex (women/men)	13 : 4	16 : 7		7 : 13	8 : 7	

Baseline data are presented as mean ± SEM, or median with interquartile. Based on the normality test, nonparametric comparison (Mann–Whitney test) or unpaired *t*-test (two-tailed) was applied to test significance between diabetic and nondiabetic subjects in both groups, *p* value ≤0.05 was considered significant (^∗^), and *p* value <0.01 indicates highly significant values (^∗∗^). SBP, DBP: Systolic and diastolic blood pressure; TC: total cholesterol; TG: triglycerides;ALT: alanine aminotransferase; AST: aspartate aminotransferase; hsCRP: high-sensitivity c-reactive protein; FPG: fasting plasma glucose; A1c: hemoglobin A1c.

**Table 2:** Clinical characteristics of the morbid-obese participants baseline, 6-month, and 1-year post-LSG.

Variable	Morbid obese with T2D			Morbid obese without T2D		
	Baseline (*n* = 11)	6 months (*n* = 11)	1 year (*n* = 11)	Baseline (*n* = 17)	6 months (*n* = 17)	1 year (*n* = 17)
BMI (kg/m^2^)	41 ± 1	31^∗∗^ ± 0.75	31^∗∗^ ± 1	44 ± 1	31 ± 1	28 ± 1
EBL (%)	—	61^∗∗^ ± 4	59^∗∗^ ± 7	—	67^∗∗^ ± 3	83^∗∗^ ± 5
EWL (%)	—	62^∗∗^ ± 4	60^∗∗^ ± 6	—	66^∗∗^ ± 4	86^∗∗^ ± 6
Body fat (%)	45 ± 1	29^∗∗^ ± 2	27^∗∗^ ± 2	47 ± 1	32^∗∗^ ± 2	26^∗∗^ ± 2
VFA (cm^2^)	200 ± 9	121^∗^ ± 6	113^∗∗^ ± 8	211 ± 6	120 ± 13	87^∗∗^ ± 8
SBP (mmHg)	127.9 ± 3.1	117.6 ± 4.5	111.1^∗∗^ ± 3.3	125.4 ± 2.6	120.5 ± 3.5	116.8 ± 3.4
DBP (mmHg)	81.1 ± 2.2	72.9^∗^ ± 2.7	68.2^∗∗^ ± 1.6	76.4 ± 1.9	72.1 ± 1.7	69.9 ± 2
Vascular age (year)	65 ± 3	55 ± 5	50^∗^ ± 5	37 ± 2	35 ± 3	34 ± 3
TC (mM)	5.02 ± 0.34	5.2 ± 0.53	5 ± 0.46	5 ± 0.1	4.7^∗∗^ ± 0.11	4.8 ± 0.14
TG (mM)	1.84 (1.09-2.8)	0.98^∗∗^ (0.7-1.4)	1.14^∗∗^ (0.6-1.2)	0.88 (0.7-1.7)	0.76^∗^ (0.6-1.01)	0.58^∗∗^ (0.44-1)
LDL (mM)	3.15 ± 0.2	3.8 ± 0.5	3.7 ± 0.4	3.1 ± 0.1	2.8 ± 0.1	2.9 ± 0.1
HDL (mM)	0.97 ± 0.07	1.2 ± 0.08	1.3^∗∗^ ± 0.12	1.3 ± 0.08	1.4 ± 0.08	1.5^∗^ ± 0.07
VLDL (mM)	0.8 ± 0.12	0.46^∗^ ± 0.07	0.37^∗∗^ ± 0.05	0.45 ± 0.05	0.33^∗^ ± 0.02	0.3^∗∗^ ± 0.03
oxLDL (mg/mL)	1.7 ± 0.17	1.58 ± 0.3	1.55 ± 0.3	0.9 ± 0.09	0.7 ± 0.06	0.8 ± 0.08
AIP (log TG/HDL)	0.27 ± 0.09	−0.03^∗∗^ ± 0.07	−0.13^∗∗^ ± 0.07	−0.11 ± 0.07	−0.26 ± 0.03	−0.36^∗∗^ ± 0.04
hsCRP (mg/dl)	1.24 ± 0.2	0.62 ± 0.2	0.39^∗∗^ ± 0.1	1.23 ± 0.1	0.47^∗^ ± 0.06	0.47^∗^ ± 0.07
Insulin (pg/mL)	2176 ± 273	608^∗∗^ ± 64	923^∗∗^ ± 186	1132 ± 147	308^∗∗^ ± 69	298^∗∗^ ± 45
c-peptide (pg/mL)	2328 ± 303	1164^∗^ ± 129	1156^∗∗^ ± 189	1804 ± 172	1063^∗^ ± 128	843^∗∗^ ± 92
FPG (mM)	10.6 ± 0.7	5.4^∗∗^ ± 0.2	5.4^∗∗^ ± 0.2	5.4 ± 0.1	4.8^∗^ ± 0.06	4.8^∗∗^ ± 0.1
A1c (%)	8.5 ± 0.5	6^∗∗^ ± 0.12	5.6^∗∗^ ± 0.1	5.7 ± 0.1	5.3^∗^ ± 0.09	5.4^∗^ ± 0.1
ALT (IU/L)	40 (37-53)	31 (28-34)	34 (29-38)	39 (32.5-53)	31 (27-34)	29 (27-39)
AST (IU/L)	23 (17-28)	15 (12-19)	18 (16-25)	18 (15.5-27)	18 (14.5-19)	13 (10-19)

Baseline, 6 months, and 1-year post-LSG data are presented as mean ± SEM, or median with interquartile. Analysis of variance (ANOVA) (unpaired analysis) with post hoc Bonferroni test for multiple comparisons was used to determine significant change post-LSG in diabetic and nondiabetic morbid-obese subjects, *p* value ≤0.05 was considered significant. (^∗^), and *p* value <0.01 indicates highly significant values (^∗∗^). VFA: visceral fat area; %EWL: percent excess weight loss; %EBL: percent of excess BMI loss.

## Data Availability

Data will only be shared upon request from the corresponding authors due to unpublished data and ethical restriction by the institute.

## Abbreviations

AIP: Atherogenic index of plasma
T2D: Type 2 diabetes mellitus
CVD: Cardiovascular disease
LSG: Laparoscopic sleeve gastrectomy
BMI: Body mass index
%EWL: Percent excess weight loss
%EWL: Percent of excess BMI loss
SBP, DBP: Systolic and diastolic blood pressure
HDL: High-density lipoprotein-cholesterol
LDL: Low-density lipoprotein-cholesterol
VLDL: Very low-density lipoprotein
oxLDL: Oxidized low-density lipoprotein
TC: Total cholesterol
TG: Triglycerides
hsCRP: High sensitivity c-reactive protein
FPG: Fasting plasma glucose
A1c: Hemoglobin A1c.

## References

[1] Grundy S. M., Cleeman J. I., Daniels S. R. (2005). Diagnosis and management of the metabolic Syndrome. *Circulation*.

[2] Eckel R. H., Grundy S. M., Zimmet P. Z. The metabolic syndrome. *The Lancet*.

[3] João Cabrera E., Valezi A. C., Delfino V. D. A., Lavado E. L., Barbosa D. S. (2010). Reduction in plasma levels of inflammatory and oxidative stress indicators after roux-en-Y gastric bypass. *Obesity Surgery*.

[4] Uzun H., Konukoglu D., Gelisgen R., Zengin K., Taskin M. (2007). Plasma protein carbonyl and thiol stress before and after laparoscopic gastric banding in morbidly obese patients. *Obesity Surgery*.

[5] Kisakol G., Guney E., Bayraktar F., Yilmaz C., Kabalak T., Özmen D. (2002). Effect of surgical weight loss on free radical and antioxidant balance: a preliminary report. *Obesity Surgery*.

[6] Nedeljkovic Z., Gokce N., Loscalzo J. (2003). Mechanisms of oxidative stress and vascular dysfunction. *Postgraduate Medical Journal*.

[7] Luc K., Schramm-Luc A., Guzik T. J., Mikolajczyk T. P. (2019). Oxidative stress and inflammatory markers in prediabetes and diabetes. *Journal of Physiology and Pharmacology*.

[8] Esteve E., Ricart W., Fernández-Real J. M. (2005). Dyslipidemia and inflammation: an evolutionary conserved mechanism. *Clinical Nutrition*.

[9] Nègre-Salvayre A., Augé N., Camaré C., Bacchetti T., Ferretti G., Salvayre R. (2017). Dual signaling evoked by oxidized LDLs in vascular cells. *Free Radical Biology & Medicine*.

[10] Rached F. H., Chapman M. J., Kontush A. (2014). An overview of the new frontiers in the treatment of atherogenic dyslipidemias. *Clinical Pharmacology and Therapeutics*.

[11] Wang J., Tan G.-J., Han L.-N., Bai Y.-Y., He M., Liu H.-B. (2017). Novel biomarkers for cardiovascular risk prediction. *Journal of Geriatric Cardiology*.

[12] Cicero A. F. G., Gitto S., Fogacci F. (2018). Fatty liver index is associated to pulse wave velocity in healthy subjects: data from the Brisighella Heart Study. *European Journal of Internal Medicine*.

[13] Cicero A. F. G., Fogacci F., Tocci G. (2020). Awareness of major cardiovascular risk factors and its relationship with markers of vascular aging: data from the Brisighella Heart Study. *Nutrition, Metabolism and Cardiovascular Diseases*.

[14] Srisawasdi P., Vanavanan S., Rochanawutanon M. (2013). Heterogeneous properties of intermediate- and low-density lipoprotein subpopulations. *Clinical Biochemistry*.

[15] Dobiasova M., Frohlich J. (2001). The plasma parameter log (TG/HDL-C) as an atherogenic index: correlation with lipoprotein particle size and esterification rate inapob-lipoprotein-depleted plasma (FERHDL). *Clinical Biochemistry*.

[16] Dobiasova M., Frohlich J., Sedova M., Cheung M. C., Brown B. G. (2011). Cholesterol esterification and atherogenic index of plasma correlate with lipoprotein size and findings on coronary angiography. *Journal of Lipid Research*.

[17] Choudhary M. K., Eraranta A., Koskela J. (2019). Atherogenic index of plasma is related to arterial stiffness but not to blood pressure in normotensive and never-treated hypertensive subjects. *Blood Pressure*.

[18] O'Rourke M. F., Staessen J. A., Vlachopoulos C., Duprez D., Plante G. E. (2002). Clinical applications of arterial stiffness; definitions and reference values. *American Journal of Hypertension*.

[19] Aroor A. R., Jia G., Sowers J. R. (2018). Cellular mechanisms underlying obesity-induced arterial stiffness. *American Journal of Physiology. Regulatory, Integrative and Comparative Physiology*.

[20] Al-Shawaf E., Al-Ozairi E., Al-Asfar F. (2018). Biphasic changes in angiopoietin-like 8 level after laparoscopic sleeve gastrectomy and type 2 diabetes remission during a 1-year follow-up. *Surgery for Obesity and Related Diseases*.

[21] Faul F., Erdfelder E., Buchner A., Lang A.-G. (2009). Statistical power analyses using G∗Power 3.1: tests for correlation and regression analyses. *Behavior Research Methods*.

[22] Faul F., Erdfelder E., Lang A.-G., Buchner A. (2007). G∗Power 3: a flexible statistical power analysis program for the social, behavioral, and biomedical sciences. *Behavior Research Methods*.

[23] American Diabetes Association (2018). 2. Classification and Diagnosis of Diabetes: *Standards of Medical Care in Diabetes—2019*. *Diabetes Care*.

[24] D’Agostino R. B., Vasan R. S., Pencina M. J. (2008). General cardiovascular risk profile for use in primary care: the Framingham Heart Study. *Circulation*.

[25] Freestone B., Krishnamoorthy S., Lip G. Y. H. (2014). Assessment of endothelial dysfunction. *Expert Review of Cardiovascular Therapy*.

[26] Icli A., Cure E., Uslu A. U. (2016). The Relationship Between Atherogenic Index and Carotid Artery Atherosclerosis in Familial Mediterranean Fever. *Angiology*.

[27] Freeman M. W., Walford G. A., Jameson J. L., Groot L. J., Kretser D. M. (2016). Chapter 41- Lipoprotein Metabolism and the Treatment of Lipid Disorders. *In: Endocrinology: Adult and Pediatric (Seventh Edition)*.

[28] Krauss R. M. (2004). Lipids and lipoproteins in patients with type 2 diabetes. *Diabetes Care*.

[29] Cure M., Tufekci A., Cure E. (2013). Low-density lipoprotein subfraction, carotid artery intima-media thickness, nitric oxide, and tumor necrosis factor alpha are associated with newly diagnosed ischemic stroke. *Annals of Indian Academy of Neurology*.

[30] Yildiz G., Duman A., Aydin H. (2013). Evaluation of association between atherogenic index of plasma and intima-media thickness of the carotid artery for subclinic atherosclerosis in patients on maintenance hemodialysis. *Hemodialysis International*.

[31] Onat A., Can G., Kaya H., Hergenc G. (2010). "Atherogenic index of plasma" (log_10_ triglyceride/high- density lipoprotein−cholesterol) predicts high blood pressure, diabetes, and vascular events. *Journal of Clinical Lipidology*.

[32] Zhu X.-W., Deng F.-Y., Lei S.-F. (2015). Meta-analysis of Atherogenic index of plasma and other lipid parameters in relation to risk of type 2 diabetes mellitus. *Primary Care Diabetes*.

[33] Wolfe B. M., Kvach E., Eckel R. H. (2016). Treatment of obesity: weight loss and bariatric surgery. *Circulation Research*.

[34] Blake G. J., Rifai N., Buring J. E., Ridker P. M. (2003). Blood pressure, C-reactive protein, and risk of future cardiovascular events. *Circulation*.

[35] Holvoet P. (2008). Association between circulating oxidized low-density lipoprotein and incidence of the metabolic syndrome. *JAMA*.

[36] Weinbrenner T., Schroder H., Escurriol V. (2006). Circulating oxidized LDL is associated with increased waist circumference independent of body mass index in men and women. *The American Journal of Clinical Nutrition*.

[37] Kutkiene S., Petrulioniene Z., Laucevicius A. (2018). Cardiovascular risk profile of patients with atherogenic dyslipidemia in middle age Lithuanian population. *Lipids in Health and Disease*.

[38] Adolphe A. B., Huang X., Cook L. S. (2011). Carotid intima-media thickness determined vascular age and the Framingham Risk Score. *Critical Pathways in Cardiology*.

[39] Jahangiry L., Farhangi M. A., Rezaei F. (2017). Framingham risk score for estimation of 10-years of cardiovascular diseases risk in patients with metabolic syndrome. *Journal of Health, Population and Nutrition*.

